# Impact of Cannabis Use on Brain Structure and Function in Suppressed HIV Infection

**Published:** 2020-08-21

**Authors:** Kalpana J. Kallianpur, Rasmus Birn, Lishomwa C. Ndhlovu, Scott A. Souza, Brooks Mitchell, Robert Paul, Dominic C. Chow, Lindsay Kohorn, Cecilia M. Shikuma

**Affiliations:** 1 Department of Tropical Medicine, Medical Microbiology and Pharmacology, University of Hawaii-Manoa, Honolulu, HI, USA; 2 Center for Translational Research on Aging, Kuakini Medical Center, Honolulu, HI, USA; 3 Hawaii Center for AIDS, University of Hawaii-Manoa, Honolulu, HI, USA; 4 Department of Medical Physics, School of Medicine and Public Health, University of Wisconsin-Madison, Madison, WI, USA; 5 Weill Cornell Medicine, New York, NY, USA; 6 The Queen’s Medical Center, Honolulu, HI, USA; 7 Missouri Institute of Mental Health, University of Missouri-St Louis, St. Louis, MO, USA

**Keywords:** Resting-State Functional Connectivity, Occipital, Atrophy, Inflammation, Monocytes

## Abstract

**Background:**

Brain atrophy and cognitive deficits persist among individuals with suppressed HIV disease. The impact of cannabis use is unknown.

**Methods:**

HIV+ and HIV− participants underwent cross-sectional magnetic resonance imaging and neuropsychological testing. Lifetime frequency, duration (years), and recency of cannabis use were self-reported. Relationships of cannabis use to resting-state functional connectivity (RSFC) and to 9 regional brain volumes were assessed with corrections for multiple comparisons. Peripheral blood cytokines and monocyte subsets were measured in the HIV+ group and examined in relation to cannabis exposure.

**Results:**

We evaluated 52 HIV+ [50.8 ± 7.1 years old; 100% on antiretroviral therapy ≥ 3 months; 83% with plasma viral load < 50 copies/mL] and 55 HIV− [54.0 ± 7.5 years old] individuals. Among HIV+ participants, recent cannabis use (within 12 months) was associated with diminished RSFC, including of occipital cortex, controlling for age. Duration of use correlated negatively with volumes of all regions (most strikingly the nucleus accumbens) independently of recent use and intracranial volume. Recent use was associated with larger caudate and white matter volumes and lower soluble vascular cell adhesion molecule-1 and monocyte chemoattractant protein-1 concentrations. Duration of use correlated positively with psychomotor speed. Use > 10 times/lifetime was linked to more somatic symptoms, better executive function, and lower CD14^+^CD16^++^ monocyte count.

**Conclusion:**

HIV+ individuals demonstrated opposing associations with cannabis. Recent use may weaken RSFC and prolonged consumption may exacerbate atrophy of the accumbens and other brain regions. More frequent or recent cannabis use may reduce the inflammation and CD14^+^CD16^++^ monocytes that facilitate HIV neuroinvasion. HIV-specific cannabis studies are necessary.

## 1. Introduction

Rates of current and lifetime substance abuse are elevated in the HIV-positive (HIV+) population [1] [2]. Use of cannabis (marijuana) is particularly prevalent [3] [4] [5]. In the U.S., 38% of HIV+ individuals reported using cannabis within the past year [6]. Recreational and medical consumption overlap, with 80% of HIV+ medicinal cannabis users also reporting recreational consumption [7]. Reasons for medicinal use include the alleviation of depression and discomfort due to combination antiretroviral therapy (cART) [4] [8] [9] [10] [11]. Cannabis use for medical purposes is currently legal in 33 states [12], while its non-medical use has been decriminalized in 16 states and legalized in 11 states and the District of Columbia [13]. Probable adverse effects of regular cannabis consumption include a dependence syndrome and impaired respiratory, cardiovascular and neuropsychological functioning [14]. Yet the drug’s cognitive consequences for HIV+ individuals remain controversial [15].

Acutely impairing effects of cannabis on cognition are well known [16]. Functional neuroimaging has revealed the acute modulation of brain function, with Δ ^9^-tetrahydrocannabinol (THC) and cannabidiol (CBD) generally having opposing neurophysiological effects [17]. Findings on the cognitive impact of chronic cannabis use are mixed [14] [16] [18] but attention and memory appear to be compromised by long-term or heavy consumption [14] [16] [19]. In HIV, cannabis may have a complicated relationship to cognitive function, depending on the pattern of use and on HIV disease stage [15] [20]-[25].

Although some studies of the general population found no brain structural differences related to chronic cannabis use [26], others have associated prolonged exposure with altered brain volumes [18]. Heavy cannabis mono-users have smaller volumes of hippocampus and amygdala [27]. HIV patients on cART may undergo progressive brain atrophy [28]. Thames, Kuhn *et al.* [21] reported that marijuana use and HIV infection were independently associated with reduced cortical volume and thickness; however, few studies have examined cannabis effects on brain structure in the presence of HIV. Investigations of brain function have also addressed cannabis use and HIV separately. THC, the main psychoactive component of cannabis [29], induces neurotoxic and structural changes in brain regions rich in cannabinoid CB1 receptors [30] [31] [32] [33]; e.g., basal ganglia, hippocampus, amygdala, and other structures involved in executive functioning [34]. Altered activation in the nucleus accumbens (NAcc) and basal ganglia of cannabis users was detected by functional magnetic resonance imaging (fMRI) [18]; higher as well as lower blood oxygenation-level dependent (BOLD) fMRI signals were measured during task performance [35] [36] [37]. The NAcc, amygdala, striatum, ventral pallidum, medial prefrontal cortex (mPFC), orbitofrontal cortex (OFC), and anterior cingulate (ACC) form part of the brain’s “reward circuitry” activated in drug addiction [38] [39] [40]. Chronic cannabis users performing normally on cognitive tasks may exhibit greater, more widespread brain activation than controls [36], or decreased activation accompanied by increased, possibly compensatory activation in adjacent regions [18] [41] [42]. Long-term exposure has been related to reduced OFC volume and to increased resting-state functional connectivity (RSFC) of the OFC network [43].

The need to understand how cannabis influences brain integrity in HIV+ individuals is underscored by BOLD fMRI studies that consistently reveal HIV-associated frontostriatal dysfunction [44]. Both HIV and cannabis affect the frontostriatal system, but interact in ways not yet understood [44] [45]. HIV enters the central nervous system (CNS) via transmigration of infected monocytes across the blood-brain barrier (BBB) [46] [47]. The viral trafficking establishes chronic neuroinflammation marked by production of pro-inflammatory cytokines and chemokines that contribute to neuronal dysfunction and the development of HIV-associated neurocognitive disorder (HAND) [47] [48] [49]. HIV infection is accompanied by expansion of non-classical (CD14^low^CD16^++^) peripheral blood monocytes [50], which secrete inflammatory cytokines [51]. Our study investigated associations between cannabis use and regional brain volumes, RSFC, and neuropsychiatric function in chronically HIV-infected individuals and an HIV-uninfected comparison group. Since HIV disease and a history of marijuana dependence have shown additive negative effects on procedural learning [25], we hypothesized an adverse impact of cannabis in our HIV+ participants. Peripheral blood inflammatory markers and monocyte subsets were also investigated in relation to cannabis use in HIV.

## 2. Methods

### 2.1. Study Design

We cross-sectionally evaluated HIV+ participants from the Hawaii Aging with HIV Cohort-Cardiovascular Disease (HAHC-CVD) study [52] and HIV-negative (HIV−) comparison subjects [53] who underwent brain magnetic resonance imaging (MRI). Inclusion criteria for the HIV+ participants required age ≥ 40 years; documented HIV infection; stable cART for at least 3 months prior to study entry; primary language of English; and ability to understand and provide informed consent. HIV− individuals were ≥40 years old, spoke English as their primary language, could provide informed consent, and were seronegative on enzyme-linked immunosorbent assay (ELISA). All participants fulfilled the same exclusion criteria: active psychosis; any uncontrolled major affective disorder; recorded loss of consciousness > 5 min; pregnancy or breastfeeding; factors precluding MRI (e.g., claustrophobia); and any past or present condition such as stroke or traumatic brain injury that was determined by the evaluating physician to introduce confounding factors [53].

Information (self-reported) on consumption of cannabis and other drugs consisted of duration (years) of use; lifetime frequency of use (0; 1 – 10; or >10 times); and whether the use was ≤12 months ago. Participants were categorized as recent users (exposure within the past 12 months), remote users (>12 months ago), or never-users. We used lifetime frequency of use to define occasional/frequent users (>10 times) and non-users (0 – 10 times). Alcohol use was assessed by the interview version of the Alcohol Use Disorders Identification Test (AUDIT). Blood specimens were obtained. For HIV+ individuals, plasma HIV RNA and CD4 cell counts were measured [53]; nadir CD4 was self-reported; and cytokine and monocyte data were obtained. Each participant gave written informed consent. The University of Hawaii Committee on Human Studies approved the study.

### 2.2. Neuroimaging

MRI was performed on a 3.0-Tesla Philips Medical Systems Achieva scanner equipped with an 8-channel head coil (InVision Imaging, Honolulu). A high-resolution anatomical volume was acquired with a sagittal T1-weighted 3D turbo field echo (T1W 3D TFE) sequence (echo time TE/repetition time TR = 3.2 ms/6.9 ms; flip angle 8°; slice thickness 1.2 mm with no gap; in-plane resolution 1.0 mm^2^; field of view 256 × 256 mm^2^; scan time = 10.2 min). Resting-state functional MRI (fMRI) echo-planar imaging (EPI) BOLD data were acquired for a participant subset, with subjects’ eyes closed and with whole-brain coverage (repetition time/echo time [TR/TE] = 1600 ms/22 ms with 262 time points; flip angle 70°; 3.5 mm isotropic voxels; 37 sagittal slices with no gap; scan time = 7.5 min).

T1-weighted data were processed with FreeSurfer as in [54]. Resting-state fMRI data were processed as follows. (Unless otherwise indicated, the AFNI software package [55] was used; names of AFNI programs are provided in parentheses.) The first 3 data points in each fMRI time series were discarded to allow the magnetization to reach a steady state. Data were then corrected for motion (3*dvolreg*), slice-time differences (3*dTshift*), and aligned to the structural T1-weighted image. The T1-weighted image was nonlinearly registered to the MNI template brain (*auto_warp.py*). The fMRI-to-structural and structural-to-template transformations were concatenated and applied to the original fMRI time series data in order to warp the fMRI data into template space. T1-weighted data were segmented into gray matter (GM), white matter (WM), and cerebrospinal fluid (CSF) using FSL’s *fast* command. The fMRI data were averaged over masks of the white matter and CSF (each eroded by 1 voxel), and the 6 realignment parameters, the average white matter, the average CSF signal, and their temporal derivatives were regressed out of the data. The data were then temporally bandpass filtered (0.01 – 0.1 Hz) and spatially smoothed by 6 mm (3*dBandpass*). Time points where the volume-to-volume motion (the Euclidean norm of the 6 realignment parameters) exceeded 0.2 mm were censored and not included in any of the analyses. A seed-based approach [56] evaluated whole-brain RSFC of regions of interest in bilateral NAcc, insula, amygdala, hippocampus, ACC, caudate nucleus and putamen. Voxel-wise whole brain measures of functional connectivity for each seed region were estimated by averaging the fMRI time series over the seed region and computing the correlation with all other brain voxels. The correlation coefficients were Fisher-Z transformed prior to group analyses.

### 2.3. Neuropsychological Testing

Participants completed the Beck Depression Inventory-I (BDI) [57] and a neuropsychological (NP) test battery that assessed cognitive domains affected by HIV [54]. A global NP z-score and composite, domain-specific z-scores (psychomotor speed, learning/memory, executive function, working memory) were derived [54]. BDI component (cognitive/affective and somatic) and total scores were computed.

### 2.4. Inflammatory Markers and Monocyte Phenotypes

We have previously described the measurement of circulating inflammatory markers [53] and blood monocyte subpopulations [58].

### 2.5. Statistical Analysis

Demographics, cannabis use variables and NP z-scores were compared between HIV+ and HIV− groups by two-tailed t-test, Mann-Whitney test, or Fisher’s exact test. Current and nadir CD4 counts and cytokine concentrations were log-transformed for normality. Univariate relationships were examined by Pearson correlation. Analysis of covariance controlling for intracranial volume (ICV) assessed differences in total volumes of the NAcc, amygdala, hippocampus, caudate, putamen, thalamus, pallidum, cortical GM and cerebral WM between HIV+ and HIV− participants.

Substance dependence may exacerbate HIV-associated brain atrophy [59]. We used multiple regression adjusting for ICV to examine the relationships of regional volumes to duration of cannabis use and to recent vs. remote use (or lifetime use frequency) in the HIV+ and HIV− groups. Associations with recent use are emphasized over associations with lifetime frequency, as use within the past year is likely more meaningful than use >10 times/lifetime. Age, current and nadir CD4 count, etc. were tested as confounders. Effects of tobacco smoking, alcohol use and polydrug use were considered in post-hoc volume analyses. Tobacco smoking was assessed by two binary variables: ever-smokers (current plus former smokers) vs. never-smokers, and current smokers vs. non-smokers (former smokers plus never-smokers). Using the first question of the AUDIT, which inquired about the past year, we categorized alcohol use as frequent (>2 times/week), occasional (up to 4 times/month), and never. Polydrug use (a binary yes/no variable) was defined as recent or remote use of one or more substances other than cannabis. For regression models, histograms of standardized residuals were checked for normality, and plots inspected to ensure that the residuals were normally distributed around the regression line. P < 0.05 was considered statistically significant. Associations between cannabis use and regional brain volumes were corrected for multiple comparisons using the Holm-Bonferroni criterion [60].

RSFC group differences (*i.e*., for HIV+ vs. HIV− participants; recent vs. remote HIV+ cannabis users; and recent vs. remote HIV− users) were assessed by a two-sample t-test controlling for age. Results were corrected for multiple comparisons using a cluster-based approach. This method runs a Monte Carlo simulation to determine the likelihood of false positives given the autocorrelation function computed from the data [61] [62].

Relationships of cannabis use variables to BDI and NP z-scores in HIV+ and HIV− individuals were also examined. Exploratory regression analyses assessed cannabis use associations with peripheral blood cytokines and monocyte sub-populations in HIV+ participants. Comparisons of recent vs. remote users did not include individuals who had never used cannabis. Never-users were included in analyses involving lifetime frequency of use.

## 3. Results

### 3.1. Study Participants

The sample consisted of 52 HIV+ and 55 HIV− participants (Table 1). Plasma viral load (HIV RNA) was undetectable (<50 copies/mL) in 43 of the HIV+ individuals; the remaining 9 participants had median (min–max) viral load of 180 (53 – 15,700) copies/mL. HIV+ individuals were younger than the HIV− group and had higher proportions of recent cannabis users and polydrug users, more years of cannabis use, poorer working memory, and higher BDI scores. Four HIV+ (3 with plasma HIV RNA < 50 copies/mL) and 11 HIV− participants had never used cannabis. Recent use of other illicit substances was not prevalent. Among HIV+ individuals, 5 were recent users of nitrates and 1 of methamphetamine; 1 HIV− participant had recently used crack cocaine (all 7 used the substances > 10 times). Those who had used cannabis (*i.e*., recent users plus remote users) comprised 48 HIV+ and 44 HIV− individuals.

Recent users reported longer duration of cannabis use than did remote users in both the HIV+ (Table 2) and HIV− (25 vs. 5 years, p = 0.002) groups. In HIV+ recent users, duration of use correlated with years of education (R = −0.43, p = 0.04). Older age correlated with longer duration of use among remote users (HIV+: R = 0.45, p = 0.03; HIV−: R = 0.45, p = 0.007). Duration of use did not correlate with years since HIV diagnosis, years on cART, or current or nadir CD4.

### 3.2. Regional Brain Volumes

Regional volumes did not differ between HIV+ and HIV− groups (p > 0.3). In multiple regression analyses controlling for age and ICV, lifetime frequency and duration of use had no effects on volumes in HIV− individuals. Among HIV+ participants, lifetime frequency of use related to amygdala volume (*β* = 0.32, p = 0.032); duration of use was associated with volumes of NAcc (*β* = −0.50, p = 0.001), amygdala (*β* = −0.44, p = 0.006), and caudate, hippocampus, cortical GM, cerebral WM (*β* ~ −0.35, p < 0.05). Relationships of duration of use to NAcc and amygdala volumes survived multiple comparison correction.

Table 3 presents regional volume comparisons between HIV+ recent and remote cannabis users, adjusted for ICV and years of cannabis use. (Inclusion of never-users did not alter the results.) Duration of use was inversely associated with all regional volumes after correction for multiple comparisons. Recent use was related to larger volumes of caudate, cerebral WM, pallidum, and amygdala; the first two associations survived Holm-Bonferroni correction. Covarying for ICV and duration of cannabis use, the adjusted means (SE) for HIV+ recent vs. HIV+ remote users were (in mm^3^) 516,095.5 (6709.2) vs. 479,459.0 (7016.7) for cerebral WM and 7574.4 (142.1) vs. 6952.6 (148.7) for the caudate nucleus. Effect sizes (partial *η*^2^) for HIV+ recent vs. remote users were 0.23 (cerebral WM) and 0.16 (caudate). Results for the caudate and cerebral WM changed very little when analyses were restricted to the 40 HIV+ users with plasma HIV RNA < 50 copies/mL or to the 39 HIV+ occasional/frequent users (of whom 29 were recent users). Regional volumes did not relate to recent use or duration of use among HIV− participants.

Tobacco smoking status (current smoking, or never vs. ever having smoked) and alcohol use did not affect regional brain volumetric associations with cannabis use in the HIV+ or HIV− groups. Adjusting for recent polydrug use made no difference, except (in Table 3) to strengthen the effects on caudate volume of recent cannabis use (*β* = 0.44, p = 0.001) and duration of use (*β* = −0.55, p < 0.00001).

### 3.3. Functional Brain Connectivity

**Figure 1.**
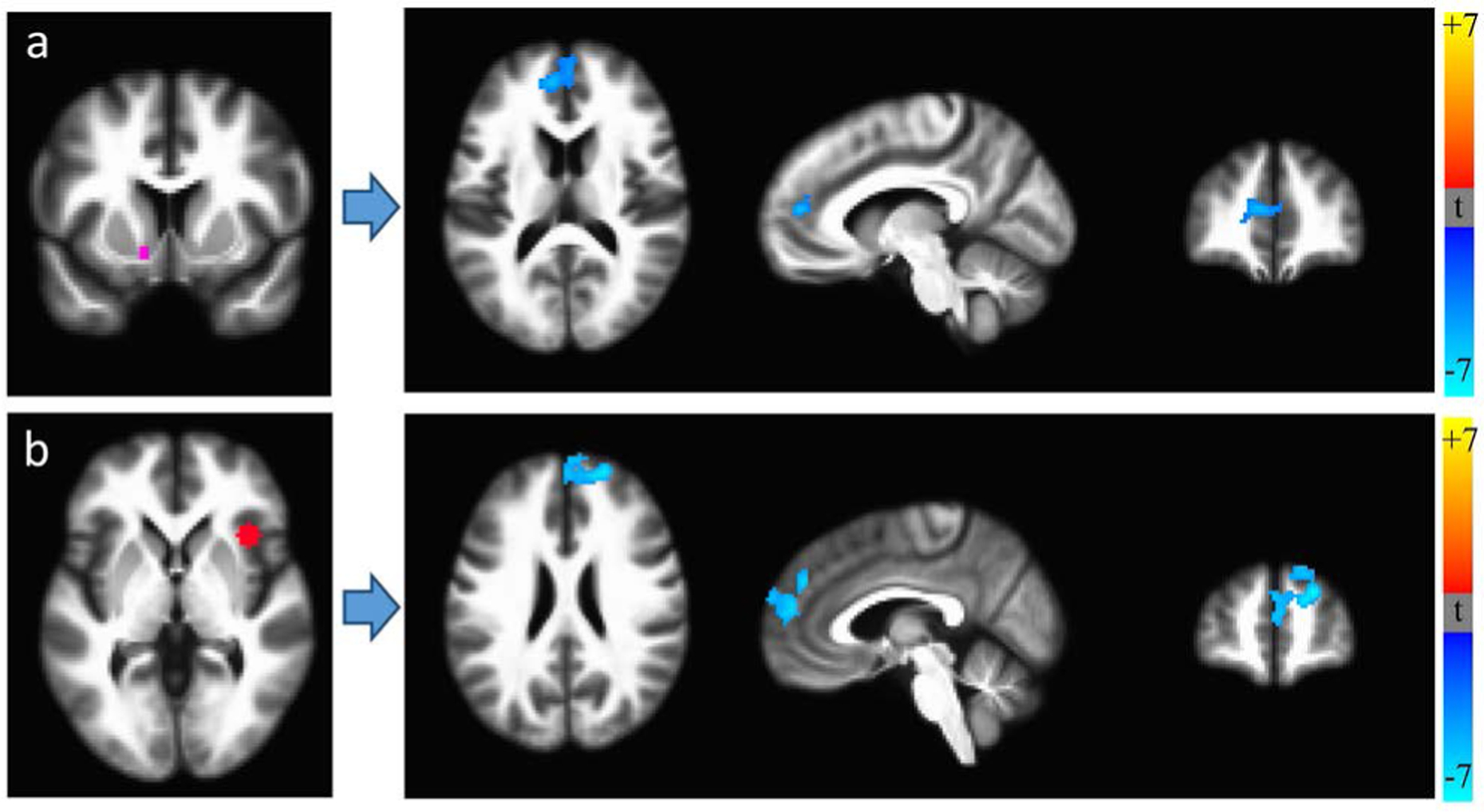
Resting-state functional connectivity differences between HIV+ (N = 21) and HIV− (N = 36) participants, controlling for age. HIV+ individuals exhibited lower RSFC between (a) a seed region in the left nucleus accumbens (*left panel*) and medial prefrontal cortex (mPFC), and (b) a seed region in the right anterior insula (*left panel*) and mPFC.

RSFC MRI scans were available for 21 HIV+ and 36 HIV− individuals (10 recent and 11 remote HIV+ cannabis users; 7 recent and 29 remote HIV− users). We first assessed RSFC differences between HIV+ and HIV− groups, applying multiple comparison corrections to achieve p < 0.05. Controlling for age, HIV+ participants had lower RSFC between the right anterior insula and mPFC and between the left NAcc and mPFC (Figure 1), consistent with task-related [63] and resting-state [64] [65] [66] decreases in frontostriatal functional connectivity in HIV.

**Figure 2.**
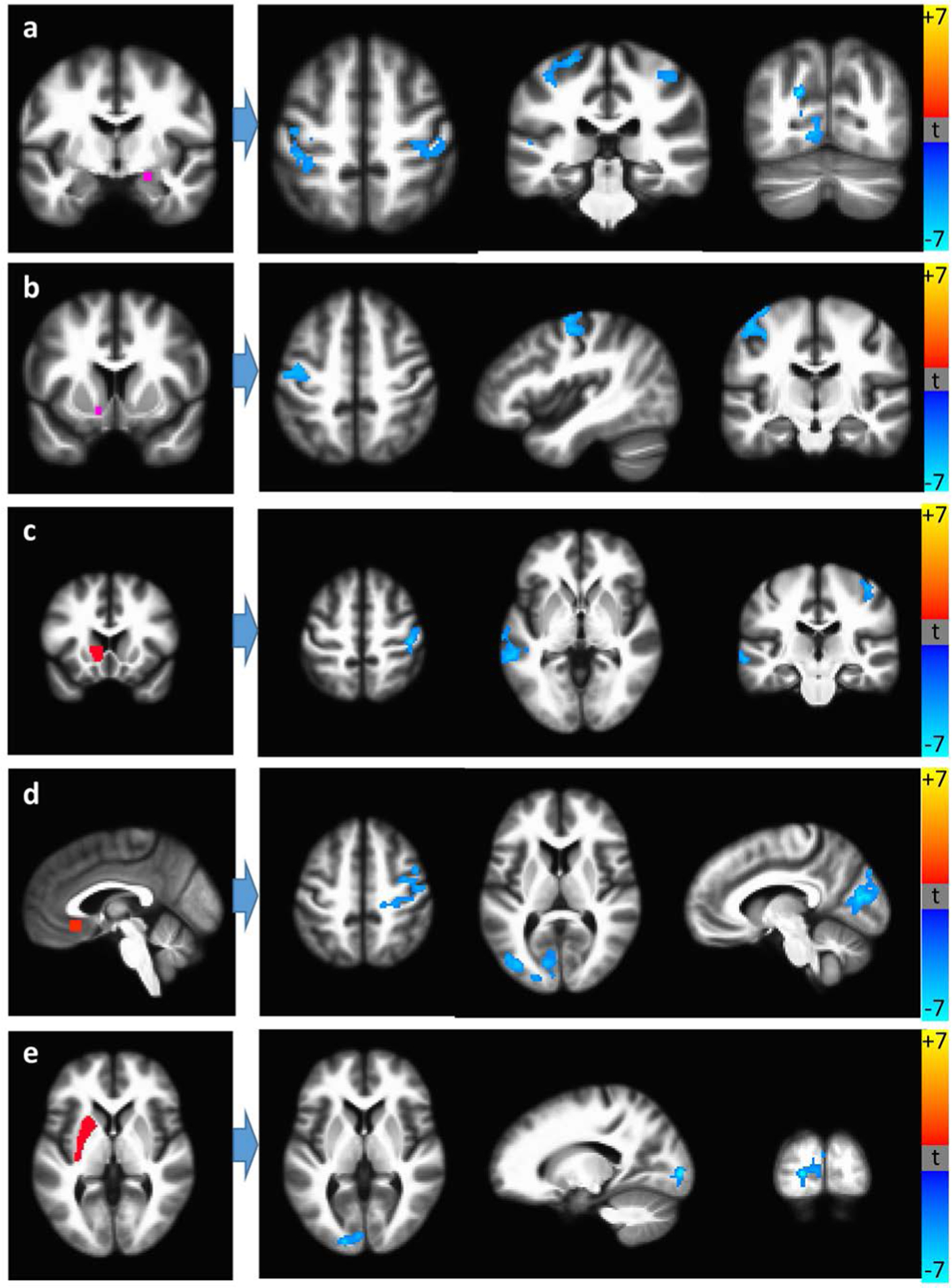
HIV+ recent cannabis users (N = 10) have lower RSFC compared to HIV+ remote users (N = 11) between (a) the right central amygdala (seed) and bilateral pre-central gyrus, left occipital cortex, central nucleus of the amygdala; (b) left nucleus accumbens (seed) and left pre-central gyrus; (c) left caudate head (seed) and right post-central gyrus, left middle temporal gyrus; (d) subgenual anterior cingulate (seed) and bilateral and medial occipital cortices, right post-central gyrus; (e) left putamen (seed) and left medial occipital cortex. Seed regions are shown on the left.

Table 4 presents significant RSFC differences between recent and remote cannabis users for the HIV+ and HIV− groups. In HIV+ participants, recent use was associated with reduced RSFC (Figure 2), including that of the occipital cortex with the amygdala, putamen and ACC. HIV+ recent users also exhibited stronger caudate-precuneus and hippocampus-motor cortex RSFC, although these results were non-significant or did not survive multiple comparison corrections. Among HIV− individuals, recent users had lower insular RSFC compared with remote users; recent use was not associated with increased RSFC.

### 3.4. Neuropsychiatric Function

HIV− recent users had lower psychomotor speed than HIV− remote users (−0.19 ± 0.75 vs. 0.48 ± 0.78; p = 0.040). Psychomotor speed did not differ between HIV+ recent vs. remote users (0.27 ± 0.65 vs. 0.24 ± 0.58; p = 0.7). Occasional/frequent use, compared to non-use, related to better executive function in HIV+ individuals (0.31 ± 0.97 vs. −0.33 ± 0.95; p = 0.046). In univariate analyses, duration of cannabis use correlated with NP z-scores for psychomotor speed (R = 0.34, p = 0.023) and executive function (R = 0.30, p = 0.045) in HIV+ participants but did not relate to NP performance in controls. When recent use and duration of use were simultaneously included as predictors in regression models, neither variable had an effect on NP z-scores for HIV− individuals; in the HIV+ group, duration of use remained associated with psychomotor speed (*β* = 0.38, p = 0.019) whereas recent use did not affect z-scores.

**Figure 3.**
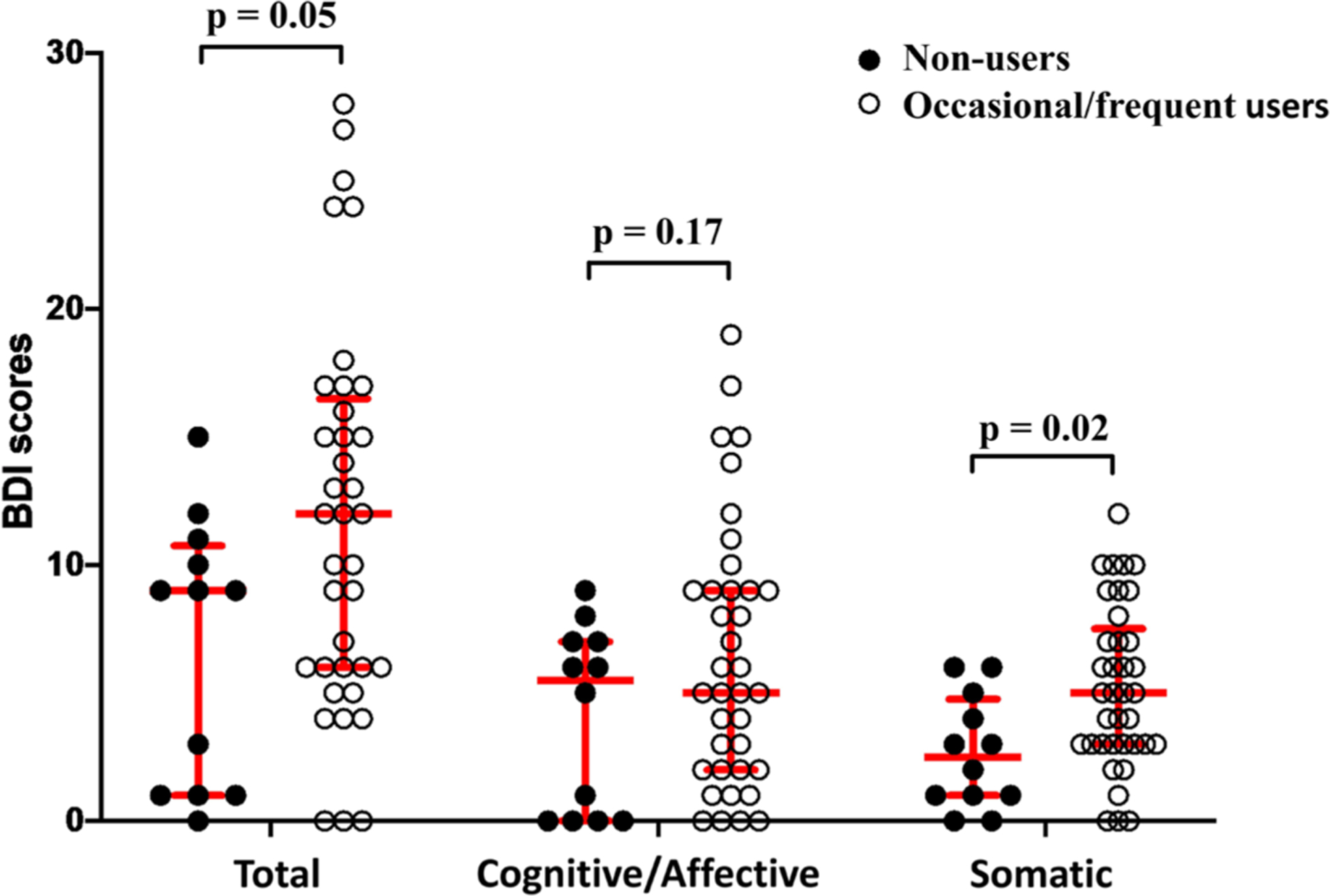
Beck Depression Inventory (BDI) somatic, BDI cognitive/affective, and total BDI scores for HIV+ occasional/frequent cannabis users (lifetime use >10 times; N = 37) and HIV+ cannabis non-users (0 − 10 times/lifetime; N = 12). Median values and interquartile range are shown.

Occasional/frequent use was associated with more somatic symptoms in HIV+ participants (Figure 3). Median (Q1 - Q3) BDI-somatic scores were 5.0 (3.0 – 7.0) for occasional/frequent users vs. 2.5 (1.0 – 4.75) for non-users (p = 0.016). Cognitive/affective BDI scores did not differ between occasional/frequent (5.0 [2.0 – 9.0]) and non-users (5.5 [0 – 7.0]; p = 0.17), but occasional/frequent users showed a trend toward higher total BDI (12.0 [6.0 – 16.5] vs. 9.0 [1.0 – 10.75]; p = 0.052). A trend association existed between higher BDI-somatic scores and recent vs. remote use (6.0 [3.0 – 9.0] vs. 3.0 [2.0 – 6.0]; p = 0.063). HIV− individuals exhibited no relationships between cannabis use and BDI.

### 3.5. Circulating Monocytes and Inflammatory Markers

Subsets of HIV+ participants had available data on plasma cytokine levels (N = 47) and absolute counts of classical, intermediate, non-classical and total monocyte populations (N = 44) (Table 5). Relative to non-users, occasional/frequent cannabis users had lower non-classical monocyte count (7.26 ± 0.36 vs. 7.51 ± 0.23 cells/L, log_10_-transformed; p = 0.048), which also related to occasional/frequent use (*β* = −0.33, p = 0.032) in regression analyses adjusting for age.

Recent use (compared to remote) was associated with lower soluble vascular cell adhesion molecule-1 (sVCAM-1) (3.00 ± 0.10 vs. 3.08 ± 0.13 pg/mL; p = 0.028) in plasma. Relative to non-users, occasional/frequent users showed a trend toward reduced sVCAM-1 (3.03 ± 0.11 vs. 3.10 ± 0.13; p = 0.073). Monocyte chemoattractant protein-1 (MCP-1) levels correlated with duration of use (R = −0.31, p = 0.039). A trend toward lower plasma MCP-1 in recent vs. remote users (2.06 ± 0.17 vs. 2.16 ± 0.18 pg/mL; p = 0.072) was significant after adjustment for age (p = 0.037).

## 4. Discussion

The apparently opposing cannabis associations demonstrated by our HIV+ study participants indicate complex effects of the drug which may be deleterious as well as beneficial. Recent users exhibited weaker brain RSFC and more somatic symptoms. Longer duration of use (reflective of prolonged or cumulative exposure) correlated with smaller volumes of multiple regions including the NAcc, hippocampus, amygdala and cortical GM. In contrast, recent use was related to slightly larger caudate and WM volumes, lower plasma MCP-1 and sVCAM-1, and better executive functioning; more frequent lifetime use was associated with lower non-classical monocyte count.

The absence of associations between cannabis use and regional brain volumes in HIV− individuals in the current study is consistent with prior findings of minimal cannabis effects, if any, on brain structure [67] [68]. However, the literature is conflicting: some non-HIV-related studies demonstrated results similar to those noted in our HIV+ group. Prolonged exposure was associated with putative brain atrophy, perhaps due to neurotoxicity of cannabis [43]. Users showed structural abnormalities in the NAcc, hippocampus, amygdala and prefrontal cortex [18] [69]. Chronic or heavy consumption was related to hippocampal and amygdalar volume reductions [18] [27] [70] [71] [72]. Moreover, addictive effects operate through increased dopamine, particularly in the NAcc [73]. As NAcc volumetric decrease is linked to apathy [74] and cognitive dysfunction [75] in chronic HIV, we find noteworthy the robust association between longer duration of cannabis use and reduced NAcc volume among our HIV+ participants. Identifying cumulative effects of cannabis on the accumbens may yield insights into the development of HIV-related mood and cognitive symptoms.

More evidence of HIV-specific cannabis effects was revealed by RSFC. Cannabis use within the past year was associated with decreased RSFC in the HIV+ and comparison groups alike; but HIV+ recent users, unlike their HIV− counterparts, demonstrated involvement of occipital cortex. HIV+ individuals exhibit visual processing/attention deficits [76] [77] and diminished activation in primary visual cortices during visual task [78] [79] [80] and resting [81] conditions. Interestingly, abnormal visual processing has distinguished HIV patients by HAND status [80]. Although our HIV+ participants did not show a relationship between cannabis use and cognitive impairment, reduced occipital RSFC in the HIV+ recent users suggests that further research is warranted on the contributions of cannabis to occipital cortical changes that potentially affect cognition.

We also observed associations between recent cannabis use and enhanced RSFC (caudate-precuneus and hippocampus-motor cortex) in the HIV+ group. These findings, while not achieving strict statistical significance, add to data indicating the complexity of cannabis effects on the brain. Functional MRI has pointed to neuroadaptive processes that may mitigate cannabis-induced impairment [18] [82]: long-term or heavy users may become tolerant to damaging cognitive effects [8] [18] [83] [84] [85]. In a non-HIV study, long-term users performed normally on cognitive control tasks but, under increasing demands, showed greater prefrontal-occipitoparietal functional connectivity (correlating with longer lifetime cannabis exposure) [82]. Duration of cannabis use has been directly correlated with RSFC [86]. While our volumetric results may imply a long-term adverse impact vs. a short-term ameliorative effect, the RSFC data illustrate the complicated nature of the consequences. A simple characterization of long-term use as detrimental is difficult to make, and in our study is contradicted by the positive relationship between duration of use and psychomotor speed in HIV+ participants [87].

Cannabis possesses anti-inflammatory properties and is under investigation for its therapeutic value [88]. It has been linked to lower HIV viral load [20]. Cannabis use in our HIV+ group was associated with decreased plasma MCP-1 and sVCAM-1, biomarkers for inflammatory processes involved in trafficking of HIV+ monocytes into the brain. Monocyte migration into the CNS requires leukocyte recruitment and adhesion to the BBB vascular endothelium, with upregulated adhesion molecules [89] [90] [91] [92]. CNS infiltration of HIV+ leukocytes is also mediated by MCP-1 [93]. MCP-1 elevations during neuroinflammation disrupt BBB integrity [94], and, in HIV, are associated with cognitive deficits [95]. Furthermore, cannabis use correlated with lower CD14^+/low^CD16^++^ monocyte counts in our HIV+ participants in agreement with published findings [96]. Non-classical monocytes, considered pro-inflammatory and patrolling [97] [98], selectively transmigrate across the BBB and facilitate HIV neuroinvasion [99] [100]. HIV+ CD14^+^CD16^+^ monocytes can differentiate into macrophages that constitute CNS viral reservoirs [100] [101]. *In vitro* THC treatment of monocytes lowers macrophage susceptibility to HIV [102] and may retard monocyte processes implicated in HIV-related neuroinflammation [96]. We found cannabis use to be associated with better neurocognitive performance in our HIV+ group, reminiscent of a positive relationship between lifetime cannabis use and verbal fluency in another HIV+ sample [87]. Reduced inflammation was recently suggested to underlie a link between cannabis exposure and lower likelihood of neurocognitive impairment in HIV [24]. Comprehensive studies are required to understand the mechanism of cannabis effects on HIV neuropathogenesis and to shed light on the interaction between cannabis consumption and HIV. The drug’s impact may well be disease-specific, given that cannabis use has been associated with better neurocognitive function in bipolar disorder but with compromised neurocognition in schizophrenia [103] [104].

It is worth noting that medical marijuana may not produce the adverse neurocognitive consequences often seen in recreational users. Individuals who sought cannabis treatment for anxiety, sleep disturbance, etc. have reported better executive functioning after three months [105]. While cognitive performance may improve solely as a consequence of symptom amelioration and concomitant lessening of other conditions (e.g., depression), medical cannabis products may be inherently neuroprotective as they are usually lower in THC and higher in CBD [105]. In our study, the product type and purpose of use were not recorded, so we cannot distinguish the possible contributions of recreational and medical cannabis to our results.

Other limitations of this work include the cross-sectional design which precludes causal inference. The lack of brain volumetric associations with cannabis use in HIV− individuals may reflect the relatively few HIV− recent users. Cannabis use information was self-reported; unknown variables included age of onset, periods of abstinence, cumulative lifetime exposure, and whether participants met Diagnostic and Statistical Manual of Mental Disorders criteria for any current substance use disorder or lifetime dependence diagnosis. Notoriously difficult to quantify, cannabis consumption presents a major challenge to research [96] [105]. Given the paucity of research on cannabis-related brain differences in HIV, our findings extend previous findings and may inform future work. Many prior neuroimaging studies of cannabis effects were restricted to dependent or heavy users and focused on the hippocampus and amygdala. Investigations of polysubstance cannabis users yield more generalizable results than studies of mono-users [106]: our HIV+ participants typified Hawaii’s HIV population. Finally, to our knowledge this is the first study to examine RSFC in the context of both cannabis use and HIV.

The current report provides evidence that cannabis use in suppressed HIV disease may exert an adverse long-term impact on the brain that competes with protective (possibly shorter-term) effects. Prolonged exposure may cause or exacerbate brain volumetric loss. Future investigations should confirm and explore the consequences of the weaker RSFC observed in our recent users. Anti-inflammatory cannabis actions may inhibit viral CNS entry, attenuate brain atrophy, and perhaps elicit compensatory RSFC mechanisms. HIV-specific research is needed as the drug’s effects may differ by HIV serostatus. Although the higher number of somatic complaints among our HIV+ frequent or recent cannabis users may signify self-medication, host and viral factors potentially associated with this outcome should be investigated. Knowledge of how cannabis may compound or counteract the damaging consequences of HIV will likely have implications for cannabinoid-based interventions. Additional research is necessary to determine interrelationships among cannabis use, cognition, and brain integrity in individuals with HIV.

## Figures and Tables

**Table 1.** Characteristics of study participants. Values are presented as N (%), mean ± s.d., or median (Q1 - Q3). Group differences were assessed by chi-squared or Fisher’s exact test (for categorical variables) and t-test (continuous variables), except where noted.

Subject characteristics	HIV-positive	HIV-negative	p-value
N	52	55	-
Male	44 (85%)	40 (73%)	0.14
Age (years)	50.8 ± 7.1	54.0 ± 7.5	0.03
Education (years)	14.4 ± 2.3	15.2 ± 2.4	0.06
Race			
Caucasian	28 (54%)	36 (65%)	
African American/Black	0 (0%)	1 (2%)	
Native Hawaiian/Pacific Islander	7 (14%)	0 (0%)	0.31
Asian	6 (11%)	6 (11%)	
More than one race	10 (19%)	12 (22%)	
Unknown	1 (2%)	0 (0%)	
Hispanic ethnicity	9 (17%)	5 (9%)	0.26
Years since HIV diagnosis	13.3 ± 6.7	-	-
Years on combination antiretroviral therapy	11.2 ± 6.0	-	-
Nadir CD4 count (cells/mm^3^), median (Q1 - Q3)	116.0 (34.0 – 251.5)	-	-
Current CD4 count (cells/mm^3^) median (Q1 - Q3)	460.5(291.5 – 594.3)	-	-
Undetectable plasma HIV RNA (<50 copies/mL)	43 (83%)	-	-
Diabetes	4 (8%)	1 (2%)	0.15
Hypertension	13 (25%)	11 (20%)	0.54
History of myocardial infarction	1 (2%)	0 (0%)	0.49
Cannabis use			
Ever used	48 (92%)	44 (80%)	0.07
Recent use	25 (52%^A^)	9 (21%^A^)	0.002
Lifetime frequency of use (0 – 10 times)	13 (25%)	23 (42%)	0.07
Lifetime frequency of use (>10 times)	39 (75%)	32 (58%)	
Duration of cannabis use (years)	10.0 (5.0 – 30.0)	6.0 (1.0 – 20.0)	0.02^‡^
Tobacco smoking			
Current smokers	17 (33%)	9 (16%)	0.05
Ever smokers	34 (65%)	34 (62%)	0.70
Alcohol Use°			
Never	20 (39%)	14 (26%)	0.09
Occasional	20 (39%)	20 (36%)	
Frequent	10 (20%)	21 (38%)	
Polydrug uset†			
Ever used	45 (87%)	37 (67%)	0.02
Recent use	8 (18%)^1^	3 (8%)^2^	0.20
Composite neuropsychological test z-scores			
Learning and memory	−0.26 ± 1.03^3^	0.09 ± 0.99	0.09
Executive function	0.15 ± 0.99	0.21 ± 1.17	0.77
Working memory	−0.14 ± 0.78	0.29 ± 0.89	0.01
Psychomotor speed	0.28 ± 0.62	0.27 ± 0.84	0.96
Global	−0.07 ± 0.56^4^	0.13 ±0.62	0.11
Beck Depression Inventory (BDI) scores			
Somatic subscore, median (Q1 - Q3)	4.0 (2.5 – 6.5)	2.0 (0 – 3.0)5	<0.000^‡^
Cognitive-affective subscore, median (Q1 - Q3)	5.0 (1.5 – 9.0)	1.0 (0 – 4.0)5	0.001^‡^
Total score, median (Q1 - Q 3)	10.0 (5.0 – 15.0)	4.0 (1.0 – 7.0)^5^	<0.000^‡^

† Use of one or more of the following: cocaine, crack, phencyclidine (PCP), heroin, crystal methamphetamine (ice), lysergic acid diethylamide (LSD), stimulants, painkillers, ecstasy, nitrates, sedatives, glue, ketamine, methadone, barbiturates.

° N = 50 for HIV+ group; ^A^recent use (vs. remote use) is defined for the 48 HIV+ and 44 HIV− participants who have used cannabis (excludes never-users).

‡ Mann-Whitney U Test;

1 N = 45;

2 N = 37;

3 N = 46;

4 N = 43;

5 N = 42.

**Table 2.** Characteristics of HIV+ recent and remote cannabis users. Values are presented as N (%), mean ± s.d., or median (Q1 - Q3). Group differences were assessed by t-test (for continuous variables) and chi-squared or Fisher’s test (categorical variables), except where noted.

Characteristics	Recent users	Remote users	p-value
N	25	23	**-**
Male	22 (88%)	19 (83%)	0.70
Age (years)	49.6 ± 6.9	51.4 ± 6.3	0.35
Education (years)	13.9 ± 2.2	14.6 ± 2.3	0.27
Caucasian race	17 (68%)	10 (44%)	0.15
Years since HIV diagnosis	13.1 ± 6.4	13.7 ± 7.4	0.79
Years on combination antiretroviral therapy	11.0 ± 5.5	11.2 ± 6.7	0.92
Nadir CD4 count (cells/mm^3^), median (Q1 - Q3)	175 (10 – 250)	85.0 (50 – 245)	0.71^‡^
Current CD4 count (cells/mm^3^), median (Q1 - Q3)	466 (281 – 641)	439 (325 – 592)	0.98^‡^
Undetectable plasma HIV RNA (<50 copies/mL)	22 (88%)	18 (78%)	0.45
Cannabis use			
Lifetime frequency of use (0 – 10 times)	1 (4%)	8 (35%)	0.01
Lifetime frequency of use (>10 times)	24 (96%)	15 (65%)	0.01
Duration of cannabis use (years)	25 (5 – 30)	8 (3 – 18)	0.01^‡^
Tobacco smoking			
Current smokers	9 (36%)	8 (35%)	>0.99
Ever smokers	19 (76%)	14 (61%)	0.35
Alcohol use			
Never	9 (36%)^1^	8 (35%)	0.95
Occasional	9 (36%)^1^	10 (44%)	
Frequent	5 (20%)^1^	5 (22%)	
Polydrug use†	23 (92%)^1^	22 (96%)	0.60
Neuropsychological test z-scores			
Learning and memory	−0.31 ± 1.00^2^	−0.28 ± 1.09^3^	0.92
Executive function	0.33 ± 0.98^1^	−0.06 ± 1.04^2^	0.20
Working memory	−0.10 ± 0.72^1^	−0.22 ± 0.87^2^	0.64
Psychomotor speed	0.27 ± 0.65^2^	0.24 ± 0.59^2^	0.89
Global	0.01 ± 0.59^4^	−0.18 ± 0.54^4^	0.30
Beck Depression Inventory (BDI) scores			
Somatic subscore, median (Q1 - Q3)	6.0 (3.0 – 9.0)^1^	3.0 (2.0 – 6.0)	0.06^‡^
Cognitive-affective subscore, median (Q1 - Q3)	6.0(1.0 – 9.0)^1^	5.0 (2.0 – 9.0)	0.48^‡^
Total score, median (Q1 - Q3)	12.0 (6.0 – 17.0)^1^	9.0 (5.0 – 15.0)	0.21^‡^

† Use of cannabis and at least one of the following at any time: cocaine, crack, phencyclidine (PCP), heroin, crystal methamphetamine (ice), lysergic acid diethylamide (LSD), stimulants, painkillers, ecstasy, nitrates, sedatives, glue, ketamine, methadone, barbiturates.

‡ Mann-Whitney U Test;

1 N = 23;

2 N = 22;

3 N = 21;

4 N = 20.

**Table 3.** Regression models showing effects of recent cannabis use (within the past 12 months) and duration (years) of use on regional brain volumes in HIV+ participants (25 recent users and 23 remote users). P-values that are significant after Holm-Bonferroni correction are shown in bold.

Brain region	Predictor variables	*β*	p	R^2^	Adjusted R^2^
Caudate nucleus	Intracranial volume	0.39	0.002		
	Years of cannabis use	−0.47	**<0.001**		
	Recent cannabis use	0.36	**0.006**		
				0.44	0.40
Pallidum	Intracranial volume	0.24	0.064		
	Years of cannabis use	−0.48	**0.001**		
	Recent cannabis use	0.35	0.012		
				0.33	0.29
Amygdala	Intracranial volume	0.28	0.033		
	Years of cannabis use	−0.46	**0.001**		
	Recent cannabis use	0.31	0.027		
				0.33	0.28
Putamen	Intracranial volume	0.30	0.028		
	Years of cannabis use	−0.38	**0.011**		
	Recent cannabis use	0.25	0.083		
				0.27	0.22
Nucleus accumbens	Intracranial volume	0.25	0.044		
	Years of cannabis use	−0.57	**<0.0001**		
	Recent cannabis use	0.23	0.084		
				0.39	0.35
Thalamus	Intracranial volume	0.47	<0.001		
	Years of cannabis use	−0.34	**0.012**		
	Recent cannabis use	0.22	0.086		
				0.39	0.35
Hippocampus	Intracranial volume	0.33	0.012		
	Years of cannabis use	−0.40	0.006		
	Recent cannabis use	0.22	0.111		
				0.31	0.26
Cortical GM	Intracranial volume	0.67	<0.0001		
	Years of cannabis use	−0.30	0.007		
	Recent cannabis use	0.04	0.691		
				0.60	0.58
Cerebral WM	Intracranial volume	0.67	<0.0001		
	Years of cannabis use	−0.37	<0.001		
	Recent cannabis use	0.32	0.001		
				0.70	0.68

GM = gray matter; WM = white matter.

**Table 4.** Seed locations and regions showing significant RSFC differences between recent and remote cannabis users, for HIV+ and HIV− study participants (controlling for age). P-values are corrected for multiple comparisons.

Brain region	Peak							Center-of-mass				Cluster size			p-value	
	X			Y		Z		X		Y	Z					
***HIV-POSITIVE PARTICIPANTS***																
*Recent < remote*																
**R. Amygdala (Ce) seed**																
L. Occipital Cortex	12			44		0		14		62	6	437			0.01	
L. Pre-central Gyrus	24			30		66		38		27	55	351			0.03	
R. Pre-central Gyrus	−36			26		58		−42		24	57	287			0.05	
**L. Caudate Head seed**																
R. Post-central Gyrus	−44			26		58		−40		22	52	377			0.02	
L. Middle Temporal Gyrus	62			38		0		61		28	−4	342			0.03	
**Subgenual ACC seed**																
Medial Occipital Cortex	6			80		14		19		83	14	1252			<0.01	
L. Occipital Cortex	30			68		−8		28		63	−10	924			<0.01	
R. Occipital Cortex	−50			78		−12		−38		70	−9	850			<0.01	
R. Post-central Gyrus	−28			26		48		−40		18	52	548			0.01	
**L. Nucleus Accumbens seed**																
L. Pre-central Gyrus	44			8		60		43		15	58	334			0.03	
**L. Putamen seed**																
Medial Occipital Cortex	18			92		2		9		93	5	299			0.05	
***HIV-NEGATIVE PARTICIPANTS***																
*Recent < remote*																
**L. Amygdala (Ce) seed**																
L. Middle Frontal Gyrus	−42			14		42		−42		24	34	597			0.01	
**L. Caudate Body seed**																
L. Medial PFC	−6			40		32		−4		40	38	297			0.05	
**R. Caudate Body seed**																
R. Medial PFC	4			70		4		4		64	6	325			0.03	
**R. Caudate Head seed**																
L. Post-central Gyrus	−60			−20		36		−58		−24	34	463			0.01	
**R. Anterior insula seed**																
L. Middle Temporal Gyrus	−62			−60		2		−56		−66	6	325			0.03	
**L. Posterior Insula seed**																
L. Superior Parietal Lobule	−18			−52		68		−28		−52	62	520			0.01	
**R. Posterior Insula seed**															
L. Inferior Parietal Lobule		−48	−46		24		−44		−52		28		460	0.01	
L. Inferior Frontal Gyrus		−42	22		−14		−40		30		−14		377	0.02	
L. Posterior Insula		−32	−20		−2		−32		−16		2		357	0.02	
L. Inferior Temporal Gyrus		−42	−6		−38		−46		−6		−34		304	0.04	
**Subgenual ACC seed**															
R. Pre-central Gyrus		54	−4		50		46		−6		50		1494	<0.01	
**L. Putamen seed**															
R. Inferior Parietal Lobule		30	−50		44		40		−48		44		326	0.03	

CE = central nucleus of the amygdala; ACC = anterior cingulate cortex; PFC = pre-frontal cortex.

**Table 5.** Plasma concentrations of inflammatory cytokines and chemokines (log10-transformed) and counts of peripheral blood monocyte populations in the HIV+ study participants. Inflammatory marker and monocyte data were available for 47 and 44 individuals, respectively, except where noted.

Cytokine/chemokine	Log_10_ Concentration (pg/mL)
sE_selectin	1.53 ± 0.25
sVCAM-1	3.05 ± 0.12
sICAM-1	2.16 ± 0.18
MMP-9	1.73 ± 0.27
MPO	1.21 ± 0.29
tPAI-1	1.97 ± 0.18
CRP	4.13 ± 0.66
SAA	4.11 ± 0.72
SAP	4.99 ± 0.39
IL-l*β*	−0.52 ± 0.03
IL-6	−0.03 ± 0.45
IL-8	0.55 ± 0.17
IL-10	0.13 ± 0.75
TNF-*α*	0.34 ± 0.41
MCP-1	2.11 ± 0.18
VEGF	1.30 ± 0.43
IFN-*γ*	−0.21 ± 0.42
NT-proBNP	0.99 ± 0.71
Monocyte population	**Log**_**10**_ **Count (cells/L)**
CD14+CD16^−^, classical	8.49 ± 0.17
CD14^+^CD16^+^, intermediate	6.83 ± 0.47
CD14^low^CD16^++^, non-classical	7.32 ± 0.35
Total monocytes^†^	8.58 ±0.18

Plasma soluble (s)E-selectin, s-vascular cell adhesion molecule (sVCAM)-1, s-intercellular adhesion molecule (sICAM)-1; matrix metalloproteinase (MMP)-9, myeloperoxidase (MPO), total plasminogen activator inhibitor type-1 (tPAI)-1, C-reactive protein (CRP), serum amyloid A (SAA), serum amyloid P (SAP); interleukin (IL)-1*β*, IL-6, IL-8, IL-10; tumor necrosis factor (TNF)-*α*, monocyte chemoattractant protein (MCP)-1, vascular endothelial growth factor (VEGF), interferon (IFN)-*γ*, N-terminal pro-brain natriuretic peptide (NT-proBNP).

† N = 52.
